# 1-stage total knee arthroplasty and proximal tibial non-union correction using 3-D planning and custom-made cutting guide

**DOI:** 10.1080/17453674.2021.1894789

**Published:** 2021-03-08

**Authors:** Andreas Kappel, Poul Torben Nielsen, Søren Kold

**Affiliations:** a Interdisciplinary Orthopaedics, Aalborg University Hospital ;; b Department of Clinical Medicine, Aalborg University , Denmark

Primary total knee arthroplasty (TKA) in the presence of extra-articular deformity and non-union is challenging (Papagelopoulos et al. 2007, Sculco et al. 2019). Bony correction can be staged prior to TKA surgery or simultaneously as a 1-stage procedure. 1-stage procedures are well described in cases with tibial deformity (Wang and Wang 2002, Xiao-Gang et al. 2012, Catonné et al. 2019) and have also been described for proximal tibial non-union (Moskal and Mann 2001, Papagelopoulos et al. 2007).

We present a new technique in which 3-dimensional (3D) planning and a custom-made cutting guide were used in a 1-stage procedure treating a complex case with posttraumatic knee osteoarthritis, proximal non-union, and deformity.

### Patient

A 67-year-old and otherwise healthy male (BMI = 29), with sequala from a complex proximal left tibial fracture (AO type 41-C3), primarily treated, 2 years previously, with a combination of internal and external fixation. Complaints were knee pain, valgus deformity, and extension deficit. ROM was from 15° extension deficit to 120° of flexion.

Preoperative radiographs and CT scan showed severe lateral joint incongruency, and proximal tibial non-union, valgus deformity, and a failed osteosynthesis with a broken plate (Figure 1).

**Figure 1.**
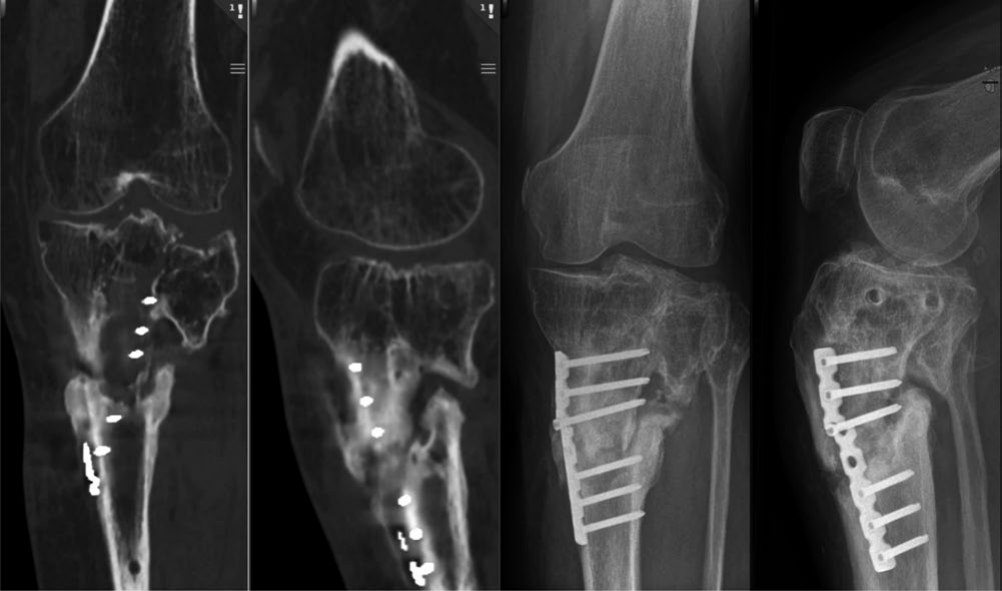
Preoperative CT and radiographs showing posttraumatic lateral tibial joint incongruence and non-union.

Treatment options included staged surgery with deformity correction and treatment of the non-union using external fixation as the first stage. Due to the joint incongruency and patient unwillingness for external fixation, we chose bony correction with closed wedge resection of non-union site and TKA in a 1-stage approach.

### Surgical planning and technique

The planning was done in cooperation with engineers from Materialise (Materialise, Leuven, Belgium). CT DICOM files were uploaded to the planning software in which resection of the non-union could be adjusted to establish optimal healthy bone contact following wedge closure. Deformity correction was planned to meet the anatomy of a statistically average model of the tibia. The program allowed visualization of resections and of the intended postoperative results with the tibial implant inserted (Figures 2 and 3).

**Figure 2.**
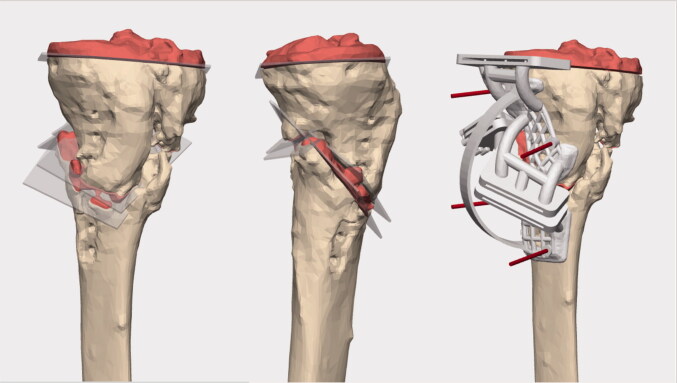
Surgical planning: red areas are planned resections, including resection of non-union site. Left: coronal view. Mid: sagittal view illustrating biplanar cuts. Right: coronal view with personalized cutting guide (Materialise planning software).

**Figure 3.**
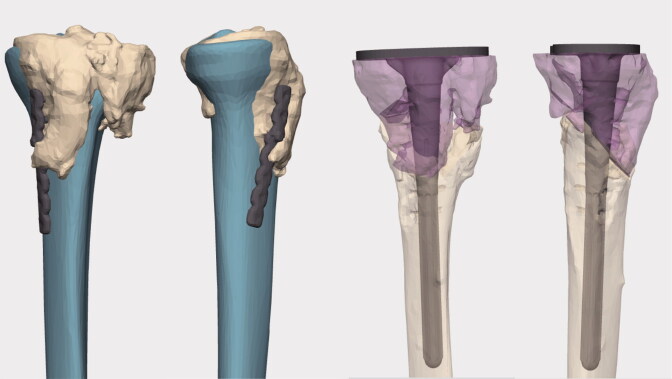
Surgical planning. Left: comparison of deformity to statistically average tibia. Right: planned correction and implant position (Materialise planning software).

A personalized cutting guide was designed to guide the resection; fit of this guide was considered essential and as minor hardware was to be removed during surgery, screw-holes from this hardware served as reference for guide placement (see Figure 3).

Surgery was performed through a standard midline incision, which was extended distally and medially to join a previous incision, a medial parapatellar arthrotomy was used, and the medial tibia was stripped from soft tissue to allow guide placement. Existing hardware was removed, and the cutting guide temporarily fixed to the bone with K-wires. Resection was performed with a reciprocating saw under fluoroscopy. Prior to closing the wedge, the resected non-union site was grafted with autologous bone graft from femoral bone cuts. TKA insertion was uneventful, the tibial component was cemented on the proximal surface and uncemented distally, and the medullary canal was reamed to obtain stability of the stem. The postoperative protocol was negative pressure wound therapy (2 weeks), mild compression bandage (6 weeks), and immediate partial weightbearing (6 weeks). Radiographs at day 1, 6 weeks, 4 months, and 1 year showed correction of the deformity with satisfying placement of the implant and bony healing (Figure 4).

**Figure 4.**
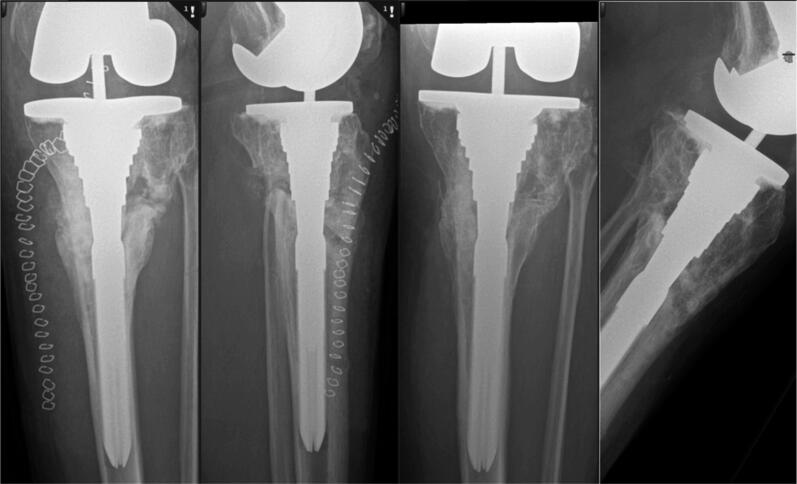
Postoperatively, planned correction and implant position obtained. Left: day 1. Right 12 months.

At 1-year follow-up there was an extension deficit of 5°; flexion was 115° Goals of pain reduction and functional improvement were met.

### Ethics, funding, and potential conflict of interests

Informed consent to publish the case was obtained from the patient. No external funding was obtained. The authors have no conflicts of interest to declare.

## Discussion

Surgical planning from a PACS viewer with inspection of 2D radiographs and 3D reconstructions was challenging in this complex case and we found the 3D planning software helpful in this process. The use of a custom guide during surgery required extended soft tissue stripping from the medial tibia; however, the guide facilitated uncomplicated resection. If resection were to have been guided by K-wires inserted under fluoroscopy we believe that only a simpler resection would have been feasible; this would have compromised the bony contact area and healing following wedge closure.

Bone union following non-union correction is dependent on stable fixation, mechanical alignment, and early functional rehabilitation (Ferreira and Marais 2015, Windolf et al. 2020). In the technique described the tibial implant is fixed to the proximal segment with surface cementing and to the distal segment with an uncemented splined stem. Stability of the construct is enhanced by bony stability obtained from the exact biplanar resection, and there is no need for other fixation of the osteotomy with plates or screws as described by previous authors (Moskal and Mann 2001).

The advantages of using these new techniques in complex cases are supported by previous reviews (Tack et al. 2016, Ejnisman et al. 2021).
